# The GEF Cytohesin-2/ARNO Mediates Resistin induced Phenotypic Switching in Vascular Smooth Muscle Cells

**DOI:** 10.1038/s41598-020-60446-z

**Published:** 2020-02-28

**Authors:** Yvonn Heun, Pascal Gräff, Aikaterini Lagara, Romina Schelhorn, Ramona Mettler, Ulrich Pohl, Hanna Mannell

**Affiliations:** 1 Institute of Cardiovascular Physiology and Pathophysiology at the Walter-Brendel-Centre of Experimental Medicine, Biomedical Center, Ludwig-Maximilians-University, Großhaderner Str. 9, 82152 Planegg, Germany; 2 Doctoral Programme Clinical Pharmacy, Hospital Pharmacy, University Hospital, LMU Munich, Marchioninistr. 15, 81337 Munich, Germany

**Keywords:** Cardiovascular diseases, Atherosclerosis, Molecular medicine

## Abstract

The pro-inflammatory adipokine resistin induces a phenotypic switch of vascular smooth muscle cells (VSMC), a process decisive for atherosclerosis, including morphological changes, increased synthetic activity, proliferation and migration. The guanine-exchange factor ARNO (Cytohesin-2) has been shown to be important for morphological changes and migration of other cell types. In this study we dissected the role of ARNO in resistin induced VSMC phenotypic switching and signalling. Firstly, treatment with the cytohesin inhibitor Secin H3 prevented the resistin mediated induction of morphological changes in VSMC. Secondly, Secin H3 treatment as well as expression of an inactive ARNO (EK) reduced resistin induced VSMC synthetic activity, as assessed by matrix metalloproteinase 2 (MMP-2) expression, as well as the migration into a wound *in vitro* compared to ARNO WT expression. Thirdly, we found ARNO to influence MMP-2 expression and migration via activation of p38 MAPK and the JNK/AP-1 pathway. Interestingly, these processes were shown to be dependent on the binding of PIP_3_, as mutation of the ARNO PH-domain inhibited VSMC migration, MMP-2 expression as well as p38 MAPK and JNK signalling. Thus, we demonstrate that ARNO is an important link in resistin dependent cell signalling leading to morphological changes, MMP-2 production and migration of VSMC.

## Introduction

Resistin is an adipokine, which is mainly expressed in peripheral blood mononuclear cells and macrophages^1,2^. Resistin levels were first found to be enhanced in serum of diabetic as well as obese mice^3^. In human, increased serum levels of resistin in diabetic and obese patients^4–6^ was furthermore shown to correlate with the accumulation of metabolic syndrome factors in type 2 diabetes^7^ as well as with other inflammatory diseases, such as atherosclerosis, rheumatic disease and sepsis^2,8^. As all these mentioned pathophysiological conditions are associated with vascular inflammation and thus an elevated risk of cardiovascular disease^9–11^, resistin has gained increasing interest as a potential biomarker^12^ and target of therapeutic interventions^8^ in the context of vascular inflammation and cardiovascular disease.

Upon stimulation with inflammatory cytokines or adipokines, vascular smooth muscle cells (VSMC) undergo a phenotypic switch from a quiescent differentiated contractile phenotype to a proliferative, migratory and synthetic dedifferentiated one^13^. The phenotypic changes of VSMC contribute to the development of atherosclerosis^14^. During this process VSMC undergo morphological changes and downregulate proteins determining the contractile phenotype, such as smooth muscle (SM) α-Actin or SM myosin heavy chain, and instead upregulate genes such as metalloproteinases (MMP), characteristic for an increased synthetic activity, important for the invasive process, i.e. proliferation and migration^13^. VSMC migration is a complex process involving remodelling of the cytoskeleton, integrins regulating adhesion by interaction with extracellular matrix (ECM) proteins and expression and secretion of proteases, such as MMP, making way for the migrating cell. Higher levels of resistin induce VSMC proliferation as well as migration^15,16^. In addition, resistin was found to induce the production of MMP from VSMC^16^. However, not much is known about which signalling intermediates are involved in resistin induced VSMC migration and MMP production.

ARNO (ARF nucleotide-binding site opener; cytohesin-2) belongs to the family of cytohesins, which are guanine nucleotide exchange factors (GEFs), activating small GTPases. ARNO has been shown to contribute to cellular adhesion and transmigration of leukocytes as well as adhesion and migration of epithelial cells, MDCK cells and preadipocytes^17–20^. Interestingly, in addition to its GEF activity, ARNO contains a pleckstrin homology (PH) domain, responsible for binding inositol phospholipids. Indeed, ARNO has been demonstrated to bind to phosphatidylinositol-3,4,5-triphosphate (PIP_3_), a membrane associated product generated by the phosphatidylinositol 3-kinase (PI3-K)^21–23^ and PI3-K has in fact been shown to be important for activation of ARNO to induce cell migration^24^. In addition, activation of ARNO has been associated with activation of the extracellular-signal regulated kinase 1/2 (ERK1/2)^25,26^ and this has been demonstrated to induce migration^19^.

Although several findings would suggest a role for ARNO also in pathological migration and tissue invasion of VSMC, it is still unknown whether ARNO plays a role in the phenotypic switch of VSMC. Moreover, an involvement of ARNO in resistin mediated signalling has not yet been studied. In fact, relatively little is known about the influence of resistin on different signalling pathways in VSMC. A closer investigation of the impact of resistin on pathways important for a phenotypic switch of VSMC is important for the deeper understanding of these pathological processes and may help in finding suitable therapeutic targets to prevent or halter the process of inflammatory cardiovascular events.

In this study, we investigated if ARNO is involved in the resistin induced VSMC phenotypic switch. We found ARNO to affect morphology, the synthetic activity and migration of VSMC induced by resistin. Furthermore, ARNO was shown to be important for resistin dependent MMP-2 production as well as migration through activation of the JNK/AP-1 pathway and p38 MAPK. Finally, ARNO GEF activity as well as its PH domain were necessary for these processes.

## Results

### Cytohesin inhibition prevents resistin mediated morphological changes in VSMC

**Figure 1:**
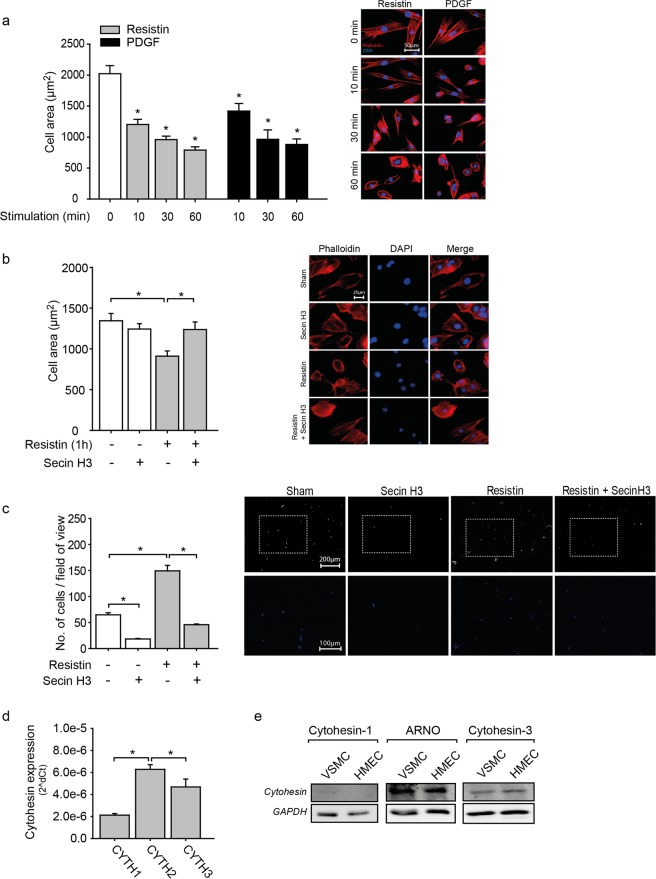
Secin H3 treatment affects the morphology of VSMC. (**a**) Treatment with pathological concentrations of resistin (100 ng/ml) caused a time dependent change in VSMC morphology (*p < 0.05 vs. non-stimulated cells, n = 6 in duplicates) similar to PDGF (10 ng/ml), which was used as a positive control (*p < 0.05 vs. non-stimulated cells, 1-way ANOVA on ranks, n = 6 in duplicates). Representative photographs are shown to the right of the graph. Morphology changes were assessed with subconfluent VSMC by staining of the actin cytoskeleton (phalloidin, red). Nuclei were stained with DAPI (blue) to visualise single cells. Bar in photo represents 50 µm. (**b**) Secin H3 treatment (15 µM^47,48^, 1 h) prevented the morphological change of VSMC induced by resistin (100 ng/ml, 1 h; *p < 0.05, 1-way ANOVA on ranks, n = 6 in duplicates). Bar in photo represents 25 µm. (**c**) Resistin treatment (100 ng/ml, 1 h) induced the adhesion of VSMC on collagen (*p < 0.05, 1-way ANOVA on ranks, n = 6 in triplicates), whereas Secin H3 treatment (15 µM, 1 h) reduced both basal and resistin induced cell adhesion (*p < 0.05, 1-way ANOVA on ranks, n = 6 in triplicates). Representative photographs of VSMC (DAPI nuclei staining, blue) are shown to the right of the graph. Bar in photo represents 200 µm. Magnifications of an area in each photo (marked by white dotted frames) are shown in the lower row (blue: DAPI). (**d**) The mRNA expression of ARNO (cytohesin-2, CYTH2) was significantly higher than cytohesin-1 (CYTH1) and cytohesin-3 (Grp-1, CYTH3) in VSMC (*p < 0.05, 1-way ANOVA on ranks, n = 12), as assessed by real-time PCR. (**e**) The protein expression of ARNO (n = 4) and cytohesin-3 (n = 3) was detected by western blot in VSMC, whereas cytohesin-1 protein expression was hardly visible (n = 3). As a positive control for ARNO expression, HMEC were used^48^. All data are presented as mean + SEM.

In their differentiated state, VSMC appear spindle shaped and long, whereas in their synthetic dedifferentiated form they have a rounder, more cobblestone like appearance^27^. First, we analysed the influence of resistin on vascular smooth muscle cell (VSMC) morphology by stimulating cells for different times with a high concentration of resistin (100 ng/ml), as this has been shown to induce an inflammatory response in VSMC and endothelial cells^28–30^, and compared it to PDGF-B stimulation, a known VSMC activator. As seen in Fig. 1a, staining with phalloidin revealed that stimulation with resistin time dependently induced the change from the elongated to a rounder shape, similar to treatment with PDGF-B. Next, we treated cells with the cytohesin inhibitor Secin H3 to investigate if cytohesins are involved in the morphological changes seen upon resistin treatment. Secin H3 treatment prevented the shape change induced by resistin (Fig. 1b). Having observed that cytohesin activity affects VSMC morphology, we next investigated its influence on VSMC adhesion to collagen. Compared to non-stimulated controls, treatment with resistin increased VSMC adhesion, which was significantly impaired by pre-treating cells with the pharmacological cytohesin inhibitor Secin H3 (Fig. 1c). Quantitative real-time PCR measurements revealed that cytohesin-1, -2 and -3 are expressed in VSMC with cytohesin-2 (ARNO), being the predominant form (Fig. 1d). Protein expression, as assessed by western blot, showed expression of ARNO and cytohesin-3, whereas cytohesin-1 was hardly detectable (Fig. 1e). Moreover, ARNO protein expression was slightly stronger upon resistin treatment (Supplementary Fig. 1). For our further studies, we decided to concentrate on the role and influence of ARNO on resistin induced effects in VSMC.

### ARNO activity influences resistin induced VSMC synthetic activity

**Figure 2:**
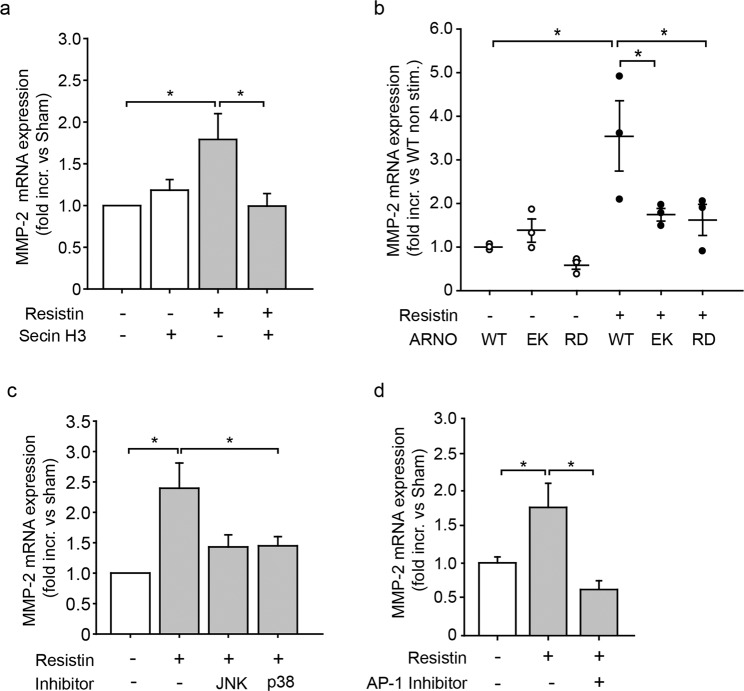
Mutation of the GEF domain or PH-domain negatively affects resistin induced MMP-2 expression. (**a**) Secin H3 treatment (15 µM) prevented the resistin (100 ng/ml, 24 h) induced expression of MMP-2 (*p < 0.05, 1-way ANOVA on ranks, n = 6–11 in duplicates). (**b**) Expression of catalytically inactive ARNO (EK) similarly inhibited the resistin (100 ng/ml, 24 h) induced MMP-2 expression in VSMC compared to ARNO WT expression (*p < 0.05, 1-way ANOVA on ranks, n = 3). Mutation of the ARNO PH-domain (RD) additionally inhibited MMP-2 expression upon resistin treatment (100 ng/ml, 24 h) (*p < 0.05, 1-way ANOVA on ranks, n = 3). (**c**) Inhibition of JNK (SP600125, 1 µM) and p38 MAPK (SB203580, 10 µM) reduced resistin (100 ng/ml, 24 h) mediated MMP-2 expression (*p < 0.05, 1-way ANOVA on ranks, n = 10 in duplicates). (**d**) Inhibition of AP-1 (SR11 302, 1 µM) prevented resistin mediated MMP-2 production (*p < 0.05, 1-way ANOVA on ranks, n = 6–11 in duplicates). All data are presented as mean + SEM.

The expression and secretion of matrix metalloproteases (MMP) mirrors the synthetic activity of dedifferentiated VSMC and play an important role in the migratory process of VSMC^14,31,32^. Treatment with pathological concentrations of resistin induced the mRNA expression of MMP-2 in VSMC, whereas ARNO inhibition by Secin H3 application prevented this (Fig. 2a). To further investigate the role of ARNO in MMP-2 production under the influence of high concentrations of resistin, an ARNO wild type (WT) construct, a catalytically inactive ARNO (by mutation of Glu at position 156 to Lys; EK)^23^ or an ARNO construct with a non-functional PH-domain (by mutation of Arg at position 280 to Asp in the PH domain; RD)^23^, all as fusion proteins with a myc-tag, were overexpressed in VSMC by pDNA transfection. The transfection efficiency was followed by detection of myc-tag and its effect on cell viability was measured by trypan blue as well as propidium iodide staining (Supplementary Fig. 2). Overexpression of ARNO EK inhibited the induction of MMP-2 mRNA expression upon resistin treatment (Fig. 2b). Interestingly, expression of ARNO lacking the ability to bind to phosphatidylinositols (RD) also prevented resistin induced MMP-2 production as compared to ARNO WT expression (Fig. 2b). The p38 mitogen activated kinase (MAPK) has been shown to influence MMP-2 production in VSMC^33^. Thus, we next treated VSMC with a pharmacological inhibitor of p38 MAPK to investigate if this kinase is involved in resistin mediated MMP-2 production. This strongly reduced the resistin induced MMP-2 expression (Fig. 2c). MMP-2 production in VSMC has in addition been shown to rely on the activator protein 1 (AP-1) transcription factor^34,35^, consisting of dimers of the c-jun, Fos or ATF proteins, which is a downstream target of the c-jun-N-terminal kinase (JNK). Treatment of VSMC with a pharmacological inhibitor of JNK or an inhibitor of the JNK downstream target AP-1 inhibited resistin induced MMP-2 expression (Fig. 2c,d).

### ARNO activity is essential for resistin induced VSMC migration

**Figure 3:**
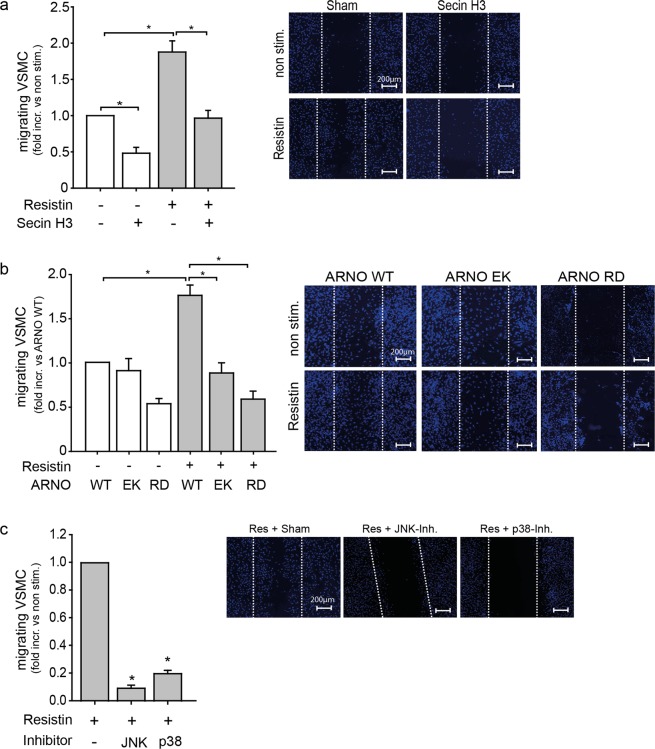
ARNO is involved in resistin induced VSMC migration. (**a**) Secin H3 treatment (15 µM) reduced basal (*p < 0.05, 1-way ANOVA on ranks, n = 11 in triplicates) and prevented resistin dependent VSMC migration into a wound *in vitro* (*p < 0.05, 1-way ANOVA on ranks, n = 11 in triplicates). (**b**) Expression of a catalytically inactive ARNO (EK) or an ARNO construct with a non-functional PH-domain (RD) prevented the resistin dependent VSMC migration compared to ARNO wildtype (WT) expressing cells (*p < 0.05, 1-way ANOVA on ranks, n = 5–6 in triplicates). (**c**) Inhibition of JNK and p38 MAPK impaired resistin (100 ng/ml) induced VSMC migration (*p < 0.05, 1-way ANOVA on ranks, n = 4 in duplicates). Representative photographs of wounded cell areas (scratches) and migrating VSMC (DAPI staining, blue) are shown to the right of all graphs. Bar in photos represents 200 µm. All data are presented as mean + SEM.

Having observed that ARNO strongly affects MMP-2 expression under inflammatory conditions, we next investigated the influence of ARNO inhibition on VSMC migration. For this, cells were stimulated with resistin and the migration into a wound (scratch) *in vitro* was evaluated. As seen in Fig. 3a, treatment with high concentrations of resistin significantly increased VSMC migration. Inhibition of ARNO activity by Secin H3 impaired both basal as well as resistin induced VSMC migration (Fig. 3a). Moreover, expression of the catalytically inactive ARNO (EK) completely inhibited VSMC migration stimulated by resistin (Fig. 3b), as compared to ARNO WT expression. As binding to PI3-K generated PIP_3_ through its PH domain has been shown to be important for ARNO activation^22,23^, we next investigated if this binding is also essential for ARNO dependent VSMC migration. Whereas resistin stimulation of VSMC overexpressing ARNO WT induced a significant migratory response, mutation of the PH-domain inhibited resistin induced VSMC migration (Fig. 3b), indicating a role of PI3-K in this context. Moreover, as we observed an impairment of resistin induced MMP-2 expression upon inhibition of the p38 MAPK and JNK/AP-1 pathways, we assessed whether these signalling pathways also influence resistin mediated VSMC migration. Treatment with p38 MAPK and JNK inhibitors additionally impaired VSMC migration (Fig. 3c).

### ARNO influences resistin induced MMP-2 expression and VSMC migration via activation of the JNK pathway and the p38 MAPK

**Figure 4:**
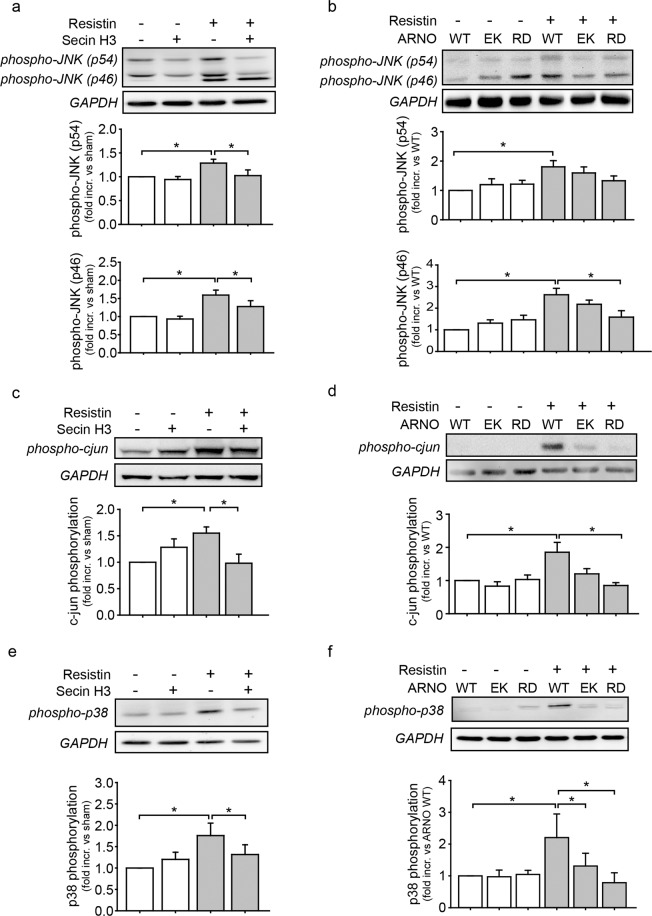
Inhibition of ARNO activity impairs resistin dependent JNK and p38 MAPK signalling. (**a**) Treatment with Secin H3 (15 µM) inhibited resistin (100 ng/ml, 10 min) mediated phosphorylation of JNK (p46 and p54, *p < 0.05, 1-way ANOVA on ranks, n = 21). (**b**) ARNO EK as well as ARNO RD expression reduced resistin induced JNK phosphorylation (p54; *p < 0.05, 1-way ANOVA on ranks, n = 17–19). (**c**) Resistin stimulation (100 ng/ml, 10 min) induced phosphorylation of the transcription factor AP-1 (c-jun) (*p < 0.05, 1-way ANOVA on ranks, n = 7) and Secin H3 (15 µM) treatment inhibited this (*p < 0.05, 1-way ANOVA on ranks, n = 7). (**d**) Expression of ARNO EK reduced and ARNO RD significantly inhibited resistin induced AP-1 activation (c-jun phosphorylation) (*p < 0.05, 1-way ANOVA on ranks, n = 12). (**e**) While resistin stimulation (100 ng/ml, 10 min) induced p38 MAPK phosphorylation (*p < 0.05, 1-way ANOVA on ranks, n = 16), Secin H3 (15 µM) treatment inhibited this (*p < 0.05, 1-way ANOVA on ranks, n = 16). (**f**) Expression of ARNO EK and ARNO RD inhibited p38 MAPK phosphorylation compared to ARNO WT expression in VSMC (*p < 0.05, 1-way ANOVA on ranks, n = 7). Quantification of protein bands normalized to GAPDH (loading control) are shown below blots. All data are presented as mean + SEM.

Having observed that ARNO affects resistin dependent migration and MMP-2 activity and that JNK and p38 MAPK inhibition impaired these processes, we next asked if ARNO influences MMP-2 expression and migration through regulation of JNK and p38 MAPK activation in VSMC. As seen in Fig. 4a, stimulation with resistin induced the phosphorylation of JNK (isoform p54 and p46) in VSMC, whereas Secin H3 treatment inhibited this. Overexpression of ARNO EK slightly and ARNO RD significantly reduced JNK phosphorylation (isoform p54) (Fig. 4b). Moreover, Secin H3 treatment inhibited resistin mediated activation of the JNK downstream target AP-1 (c-jun) (Fig. 4c). Additionally, whereas resistin induced the c-jun phosphorylation in ARNO WT expressing cells (Fig. 4d), ARNO EK as well as ARNO RD expressing cells showed impaired c-jun phosphorylation (Fig. 4d). Furthermore, Secin H3 treatment inhibited resistin mediated p38 MAPK phosphorylation (Fig. 4e). Likewise, expression of ARNO EK or ARNO RD abolished the resistin induced phosphorylation of p38 MAPK (Fig. 4f).

## Discussion

Phenotypic switching of VSMC, resulting in their proliferation and migration, is involved in the vascular response to inflammatory stimuli^36^ and the changes in VSMC function from a contractile immobile to a synthetic invasive form is believed to contribute to the development of atherosclerosis^13,14^. Although the pro-inflammatory adipokine resistin induces the proliferation and migration of VSMC^8^ and is associated with cardiovascular disease^8^, little is known about the actual signalling elicited by resistin in VSMC leading to migration. In this study we demonstrate the importance of the GEF Cytoshesin-2/ARNO for the inflammatory response of VSMC to resistin.

Treatment of resistin induced the migration of VSMC *in vitro*. Our findings are in accordance to the study of Ding *et al*.^16^, who showed that VSMC migration is induced by resistin involving PKCε. Here, we identified additional pathways activated by resistin stimulation, which were involved in the migratory response of these cells. p38 MAPK as well as JNK and its downstream target c-jun were phosphorylated upon resistin stimulation and inhibition of these kinases impaired resistin induced VSMC migration.

Of note, we discovered ARNO to be important in resistin mediated signalling and migration in VSMC, as overexpression of a GEF inactive ARNO mutant impaired activation of p38 MAPK as well as the JNK/AP-1 pathway and the migratory response. These findings are supported by the observation that treatment with the cytohesin inhibitor Secin H3 also diminished VSMC migration. Furthermore, we observed p38 MAPK and JNK/AP-1 to mediate resistin dependent MMP-2 expression. Interestingly, ARNO was in addition shown to promote resistin induced MMP-2 expression, as mutation of the Sec-7 domain, harbouring the GEF activity, as well as Secin H3 treatment reduced this. Previous studies demonstrate ARNO to drive migration by activation of the ADP-ribosylation factor GTPases ARF6 and ARL4D^18–20^. The activation of ARF6 was shown to be important for Rac as well as Dynamin activation, proteins known to regulate cell migration^18,19,24^. Moreover, by interacting with the cytoskeletal adaptor protein Paxillin, ARNO was shown to influence preadipocyte migration^37^. Although we did not investigate direct targets of ARNO, it is likely that ARF6-Rac signalling is also involved in the resistin mediated VSMC migration and morphology changes observed in this study. Our findings highlight for the first time that ARNO is important for resistin induced VSMC migration by mediating MMP-2 expression through activation of p38 MAPK and the JNK/AP-1 pathway. Indeed, c-jun and the complex AP-1 has been shown to be responsible for serum stimulated VSMC migration and MMP-2 production before^34,35,38^. Our data now show that ARNO is an important link in resistin induced signalling resulting in VSMC migration.

The PH-domain of ARNO has been shown to be important for ARNO activity^39^. Moreover, ARNO was shown to bind to PIP_3_ through its PH-domain and in this way be recruited to the cell membrane^21,22^, demonstrating a regulatory role for PI3-K in ARNO activation. In our hands, mutation of the PH-domain negatively affected resistin induced signalling, MMP-2 production and migration of VSMC, strongly arguing for a dependence of PI3-K for these processes. Indeed, PI3-K is known to be important for cell migration in general^40^ and was shown to also influence MMP-2 production in VSMC^31^. Thus, our data are in accordance to other studies, observing an important role of the PH-domain of ARNO for its cellular functions.

In addition, we observed cytohesins, particularly ARNO due to its high expression in VSMC, to influence the morphology of VSMC. ARNO has been shown to influence the morphology of epithelial cells by regulating Rac and phospholipase D activity^18^ and to be involved in cytoskeletal rearrangements^20,41^, resulting in migration. Therefore, our findings clearly demonstrate that ARNO activity likewise drives morphological and phenotypical changes in VSMC promoting migration. This may indeed have profound impact on VSMC pathophysiology, thus affecting vascular pathophysiology. Of note, our data highlight the involvement of ARNO in vascular inflammatory responses for the first time and shed light on resistin dependent signalling in VSMC. The strength of the impact of our findings in a clinical setting, however, still needs to be confirmed *in vivo* and in patient samples.

In summary, we show for the first time that ARNO is involved in resistin dependent cell signalling leading to MMP-2 production and migration of VSMC via activation of the p38 MAPK and JNK/AP-1 pathway. Furthermore, ARNO promotes morphological and phenotypical changes of VSMC upon resistin stimulation, processes known to strongly influence cell migration. Moreover, not only the GEF activity of ARNO is important for eliciting these responses, but also the PH domain, suggesting a dependency of PI3-K in these processes. As inhibition of ARNO impaired the phenotypic switch of VSMC, this GEF may become an interesting target when developing new strategies aiming at limiting the invasion of VSMC in the vasculature, such as in restenosis upon stenting or even atherosclerosis in patients prone to vascular inflammatory disease, such as diabetes or metabolic syndrome.

## Materials and Methods

### Antibodies and chemicals

Rabbit phospho-p38 MAPK (Thr180/Tyr182) (D3F9) XP^®^ (#4511), rabbit phospho-SAPK/JNK (Thr183/Tyr185) (81E11) (#4668), rabbit phospho-cjun (Ser73) (D47G9) XP^®^ (#3270) antibodies were from Cell Signaling Technology. Mouse GAPDH (#MAB374) as well as anti-mouse and rabbit horseradish peroxidase-conjugated secondary antibodies (#401253 and #401353) were from Merck Millipore. Mouse PSCD2 (ARNO) clone 6H5 antibody was from Sigma-Aldrich, and goat GRP1 (cytohesin-3) (C-20) (sc-9732) and goat cytohesin-1 (N-19) (sc-9733) antibodies were from Santa Cruz. Inhibitors for AP-1 (SR11302), JNK (SP600125), p38 MAPK (SB203580) were from Tocris. Alexa-Fluor 546 labelled Phalloidin was from Molecular Probes. Recombinant human Resistin (#RD 172016301) was from BioVendor. Recombinant human PDGF-BB (#3201) and all other chemicals were from Sigma-Aldrich.

### Cell isolation and culture

Primary porcine vascular smooth muscle cells (VSMC) were isolated and cultured from rest materials (porcine aortas) collected from a slaughter house as previously described^42^ and used up to passage five. Thus, no ethical approval was needed. The VSMC identity was verified by staining for smooth muscle α-Actin. VSMC were always seeded onto collagen G (10 µg/ml) (Biochrom) prior to any experiment. Cells were starved in DMEM containing 1% fetal calf serum (FCS), and 1% antibiotic antimycotic solution (APS, Sigma-Aldrich) containing streptomycin and amphotericin B 24 h before treatment with resistin (100 ng/ml) (BioVendor).

Human microvascular endothelial cells (HMEC) were provided by Ades *et al*.^43^ and cultured as previously described^44^.

### Plasmids and transfections

Plasmids for myc-tagged ARNO wild type (WT) and myc-tagged ARNO E156K (EK) mutant (mutation of Glu at position 156 to Lys) as well as ARNO R280D (RD) mutant (mutation of Arg at position 280 to Asp) were kindly provided by Dr. Santy^23^. VSMC were transfected with the different ARNO constructs using jetPRIME transfection reagent (PeqLab) according to manufacturer´s protocol. Cells were used for further experiments 48 h after transfection.

### Analysis of morphological changes

Subconfluent, starved (24 h) VSMC were stimulated with resistin (100 ng/ml) or PDGF (10 ng/ml) for indicated time points. Secin H3 (15 µM) or sham solution (DMSO v/v) were added 30 min prior to stimulation. The cells were then washed once in phosphate buffered saline (PBS) supplemented with calcium and fixed with 1% formalin for 10 min. at RT. After washing with PBS cells were permeabilized with 0.1% Triton X-100 for 2 min. and blocked with 1% bovine serum albumin (1 h, RT) in PBS and stained with phalloidin-AF546, to visualize F-actin, and DAPI (1 µg/ml), to visualize nuclei, (20 min, RT in the dark). After three wash steps with blocking buffer (1% bovine serum albumin in PBS), cells were imaged with an Axiovert 200 M fluorescence microscope (Zeiss) using 40x objective. The cell area (determined by phalloidin staining) was measured using the AxioVision 4.9 programme (Zeiss).

### Immunoblotting

For western blotting VSMC were washed with ice-cold PBS and subsequently lysed in ice-cold lysis buffer from Cell Signaling Technology supplemented with PSMF (20 µM). Cell lysates were prepared and subjected to SDS-PAGE and western blotting as described before^45^. Quantification of protein expression levels were performed by band intensity measurements and normalized to GAPDH band intensity using the Hokawo software (Hamamatsu).

### RNA isolation and qRT-PCR

Isolation of total RNA from VSMC was performed with the peqGOLD total RNA Kit (VWR) according to manufacturer´s description. The TaqMan real-time PCR system (Applied Biosystems) was used to perform quantitative reverse-transcriptase PCR (qRT-PCR) as previously described^46^. cDNA was produced using the High-Capacity RNA-to-cDNA™ Kit (Applied Biosystems). The following primers were used: MMP-2 Fw agg atg gca agt acg gct tc, MMP-2 Rev agc tgt tgt agg atg tgc cc; CYTH1 Fw ttt gct cac ttt tgc gcc tg, CYTH1 Rev gag gca gga gtg ctg ttc tt; CYTH2 Fw gag gac ggt gtc tac gag c, CYTH2 Rev ctc gac ttc gct cat ggc tt; CYTH3 Fw aga caa gcc agt gtc gtc ag, CYTH3 Rev tcc tgt tcg gac tcc gtt tg; s18 rRNA Fw cgc ggt tct att ttg ttg gt, s18 rRNA Rev agt cgg cat cgt tta tgg tc.

### Migration assay

The VSMC migration was measured using an *in vitro* wound healing model (scratch assay).

VSMC were grown to confluency on collagen coated cell culture dishes (24-well plate) followed by scratching the cell layer with a 0.5 mm pipet tip from edge to edge of the culture well. After scratching, the cell culture media was exchanged and starvation media containing sham (DMSO v/v) or treatment was added. Cells were left to migrate into the scratch for 16 h and subsequently fixed in 1% formalin 10 min at room temperature (RT) followed by careful rinsing in PBS supplemented with calcium. For evaluation of migration, the number of cells in the wound area (scratch) were counted after permeabilisation with 0.2% Triton X-100 in PBS and subsequent staining with DAPI (1 µg/ml, 10 min, RT) and subsequent rinsing in PBS supplemented with calcium. Fluorescent images were taken with an Axiovert 200 M microscope (Zeiss).

### Adhesion assay

VSMC were incubated in starvation media (DMEM with 1% fetal calf serum and 1% penicillin/streptomycin/antimycoticum) 24 h before seeding onto collagen G coated 8-well IBIDI slides (IBIDI). Treatments were performed immediately in starvation media and cells left to adhere for 1 h at 37 °C and 5% CO_2_. For visualisation of adhered cells, they were fixed in ice-cold methanol (70%), 30 min at −20 °C followed by washing with PBS supplemented with calcium and staining with DAPI (1 µg/ml, 10 min, RT). After rinsing in PBS supplemented with calcium fluorescent images were taken with an Axiovert 200 M microscope (Zeiss) and number of adhered cells/visual field were counted.

### Statistical analysis

Data are presented as means ± SEM and statistical analyses were performed using Sigma Plot 10.0. For multiple comparisons of normal distributed data the one-way analysis of variance (1-way ANOVA), followed by Student Newman-Keuls post-hoc test was performed. For multiple comparisons of data following a non-normal distribution, the one-way analysis of variance on ranks (Kruskal-Wallis ANOVA on Ranks) followed by either Dunn’s Method or Student Newman-Keuls was performed. Differences were considered significant at an error probability level of p < 0.05. Figures were made using Sigma Plot 10.0 and Adobe illustrator CS5.

## Supplementary information

Supplementary information

## Data Availability

The data sets generated during/or analysed during the current study are available from the corresponding author on reasonable request.
