# Supplementary material for: The GEF Cytohesin-2/ARNO Mediates Resistin induced Phenotypic Switching in Vascular Smooth Muscle Cells
Source: Sci Rep. 2020 Feb 28;10:3672. doi: 10.1038/s41598-020-60446-z (PMC7048779; doi:10.1038/s41598-020-60446-z)

The GEF Cytohesin-2/ARNO Mediates Resistin induced Phenotypic Switching in Vascular Smooth Muscle Cells

Supplementary Information

Yvonn Heun[1*], Pascal Gräff[1*], Aikaterini Lagara[1], Romina Schelhorn[1], Ramona Mettler[1], Ulrich Pohl[1], Hanna Mannell[1, 2]

[1]Institute of Cardiovascular Physiology and Pathophysiology at the Walter-Brendel-Centre of Experimental Medicine, Biomedical Center, Ludwig-Maximilians-University, Großhaderner Str. 9, 82152 Planegg, Germany. [2]Doctoral Programme Clinical Pharmacy, Hospital Pharmacy, University Hospital, LMU Munich, Marchioninistr. 15, 81337 Munich, Germany.

[*]These authors contributed equally to the manuscript.

Corresponding author

Dr. Hanna Mannell

Institute of Cardiovascular Physiology and Pathophysiology at the Walter-Brendel-Centre of Experimental Medicine, Biomedical Center, Ludwig-Maximilians-University,

Großhaderner Str. 9, 82152 Planegg, Germany

Tel: +49-89-4400-44560

Email: hanna.mannell@med.uni-muenchen.de

Supplementary materials and methods

Materials

Mouse cytohesin-2 (H-7) (sc-374640) was purchased from Santa Cruz Biotechnology. Rabbit myc-tag antibody (#2278) was from Cell Signaling Technology.

Measurement of propidium iodide uptake

For measurement of propidium iodide uptake, VSMC were gently detached from the cell dishes using 5mM EDTA followed by centrifugation (3000 rpm, 2 min) and removal of the supernatant. Cell pellets were washed and dissolved in propidium iodide staining buffer (1.2 µg/ml propidium iodide in PBS supplemented with calcium). After incubation at RT for 20 min, cells were centrifuged (3000 rpm, 2 min) and pellets dissolved in PBS supplemented with calcium followed by measurement of propidium iodide positive cells using a FACS Canto II (BD Biosciences). Positive controls were generated by incubation of fixed cells (1% formalin) in 0.1% triton X-100 to permeabilize the cell membrane prior to staining.

Trypan blue staining

VSMC were gently detached from the cell dishes using 5mM EDTA followed by centrifugation (3000 rpm, 2 min) and removal of the supernatant. Cell pellets were dissolved in trypan blue staining solution (0.1 % trypan blue in PBS supplemented with calcium) and incubated in RT for 1 min. The number of stained (blue) cells as well as the total number of cells were assessed with fast-read 102 cell counting chambers (Biosigma Srl) and the percentage of non-stained cells was calculated.

Supplementary figure legends

Supplementary Fig. 1 ARNO protein expression upon resistin treatment

ARNO protein expression in VSMC was slightly increased upon resistin treatment (100ng/ml, 24h) compared to non-treated cells (0h) and cells incubated in growth medium containing 10% FCS (*$p<0.05$, n=6, 1-way ANOVA on ranks), as assessed by western blot. Data are presented as mean + SEM.

Supplementary Fig. 2 Overexpression of ARNO constructs in VSMC

a) The expression of ARNO WT, EK and RD was verified by staining for the fusion-tag myc. b) Transfection with ARNO WT, EK or RD did not influence survival of VSMC, as assessed by trypan blue staining (n=5 in triplicates). c) This was confirmed by detection of PI-staining of transfected cells with flow cytometry (n=2, each 10.000 cells). Non transfected cells permeabilised with Triton X-100 prior to staining were used as positive control. Data are presented as mean + SEM.

Supplementary Fig. 3 Whole size western blots – detection of phosphorylated JNK

a) Phosphorylation of JNK was detected in VSMC upon Secin H3 treatment under basal conditions as well as upon resistin treatment. Lanes 7-12 are displayed in Figure 4a. b) Detection of GAPDH on the same blot as in a. c) Phosphorylation of JNK was detected in VSMC overexpressing ARNO WT, EK and RD under basal conditions as well as upon resistin treatment. Part of the blot is shown in Figure 4b. d) Detection of GAPDH on the same blot as in c.

Supplementary Fig. 4 Whole size western blots – detection of phosphorylated c-jun

a) Phosphorylation of c-jun was detected in VSMC upon Secin H3 treatment under basal conditions as well as upon resistin treatment. Part of the blot is shown in Figure 4c. b) Detection

of GAPDH on the same blot as in a. c) Phosphorylation of c-jun was detected in VSMC overexpressing ARNO WT, EK and RD under basal conditions as well as upon resistin treatment. Part of the blot is shown in Figure 4d. d) Detection of GAPDH on the same blot as in c.

Supplementary Fig. 5 Whole size western blots – detection of phosphorylated p38 MAPK

a) Phosphorylation of p38 MAPK was detected in VSMC upon Secin H3 treatment under basal conditions as well as upon resistin treatment. Part of the blot is shown in Figure 4e. b) Detection of GAPDH on the same blot as in a. c) Phosphorylation of p38 MAPK was detected in VSMC overexpressing ARNO WT, EK and RD under basal conditions as well as upon resistin treatment. Part of the blot is shown in Figure 4f. d) Detection of GAPDH on the same blot as in c.

Supplementary Fig. 6 Whole size western blots – detection of cytohesins

a) Cytohesin-1 (46 kDa) could not be detected in HMEC and VSMC. Part of the blot is shown in Figure 1e. b) Detection of GAPDH on the same blot as in a. c) ARNO (cytohesin-2) (46 kDa) was detected in VSMC and HMEC. Part of the blot is shown in Figure 1e. d) Detection of GAPDH on the same blot as in c. e) Detection of cytohesin-3 in HMEC and VSMC. Part of the blot is shown in Figure 1e. f) Detection of GAPDH on the same blot as in e. g) Detection of ARNO (46kDa) upon resistin treatment. Part of the blot is shown in supplementary Figure 1. h) Detection of GAPDH on the same blot as in g.

Supplementary figure 1

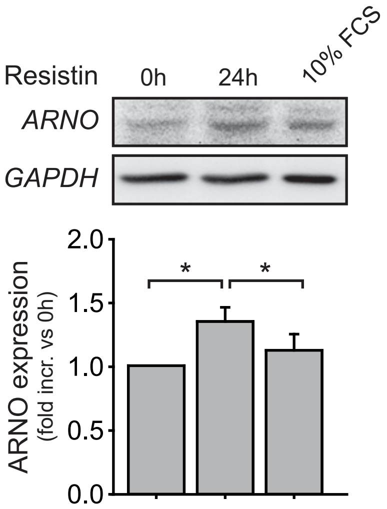

Supplementary figure 2

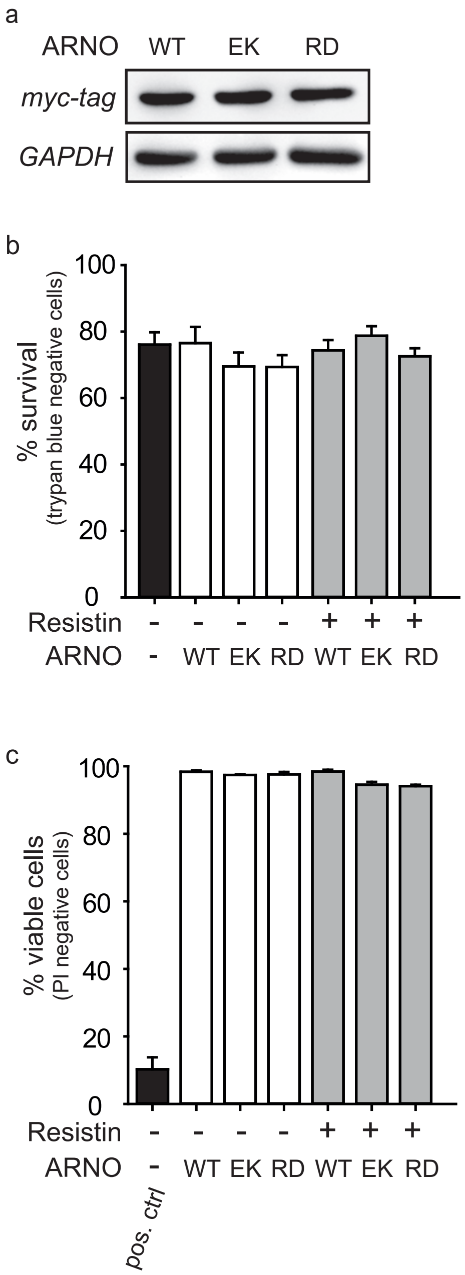

Supplementary figure 3

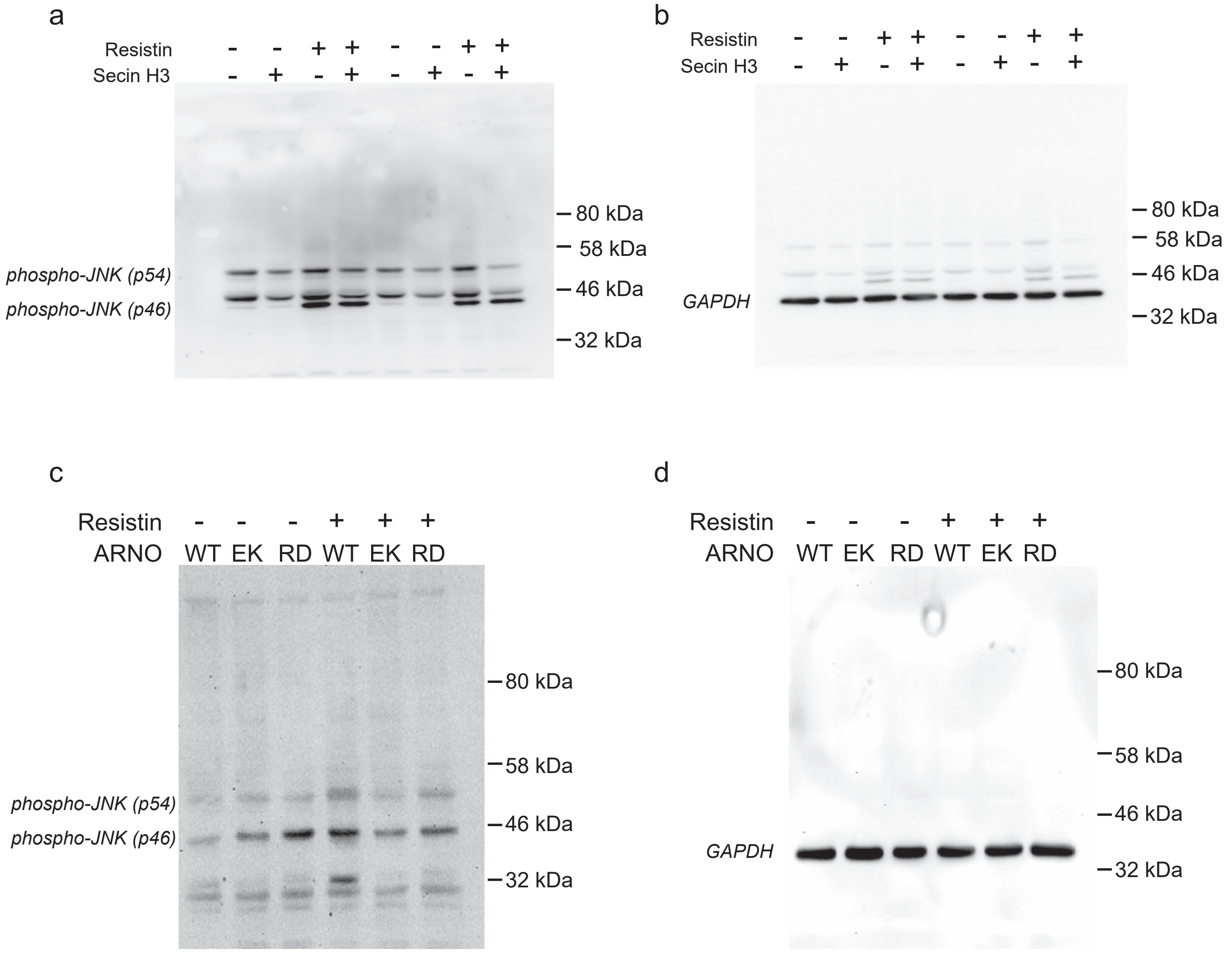

Supplementary figure 4

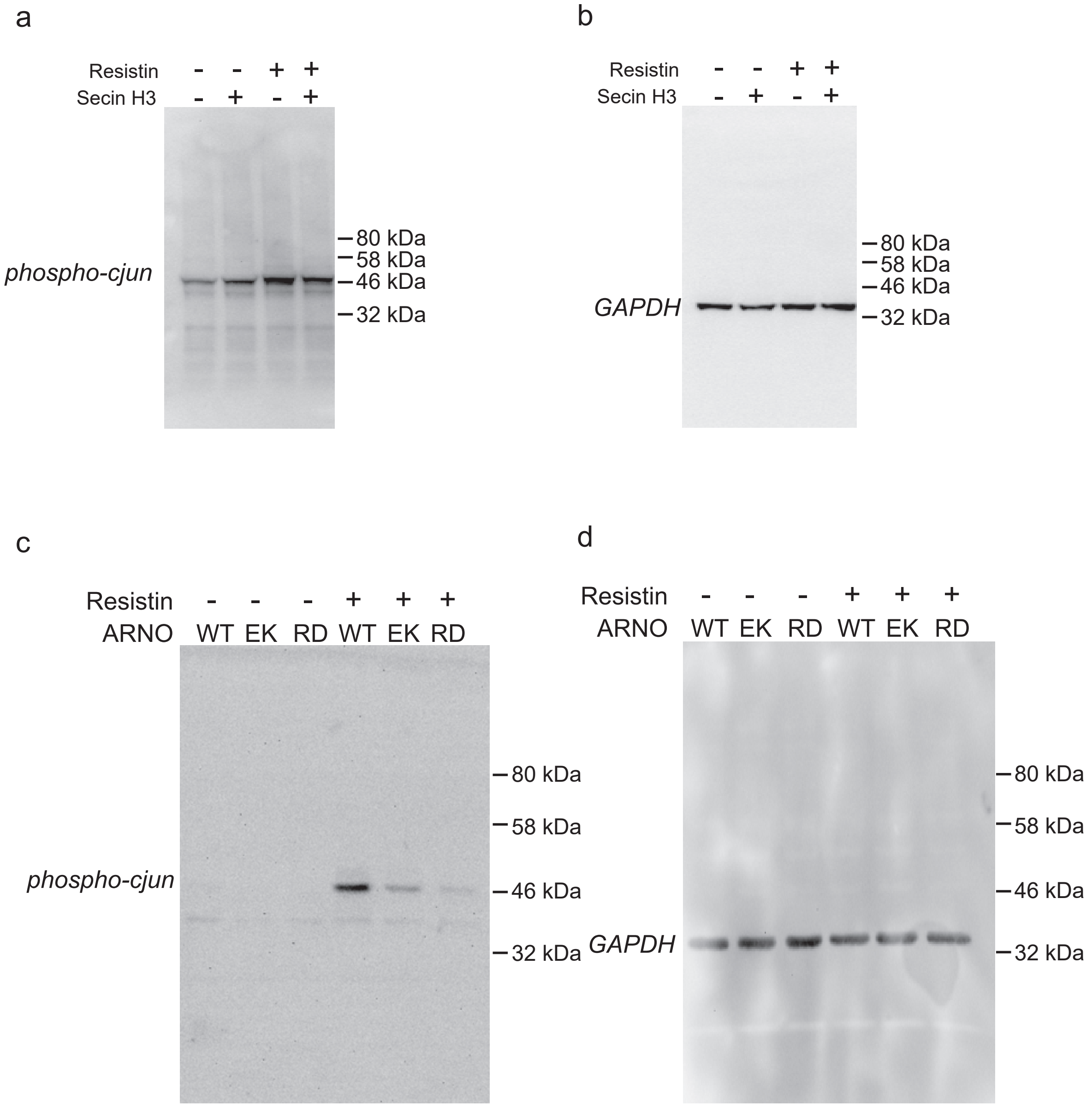

Supplementary figure 5

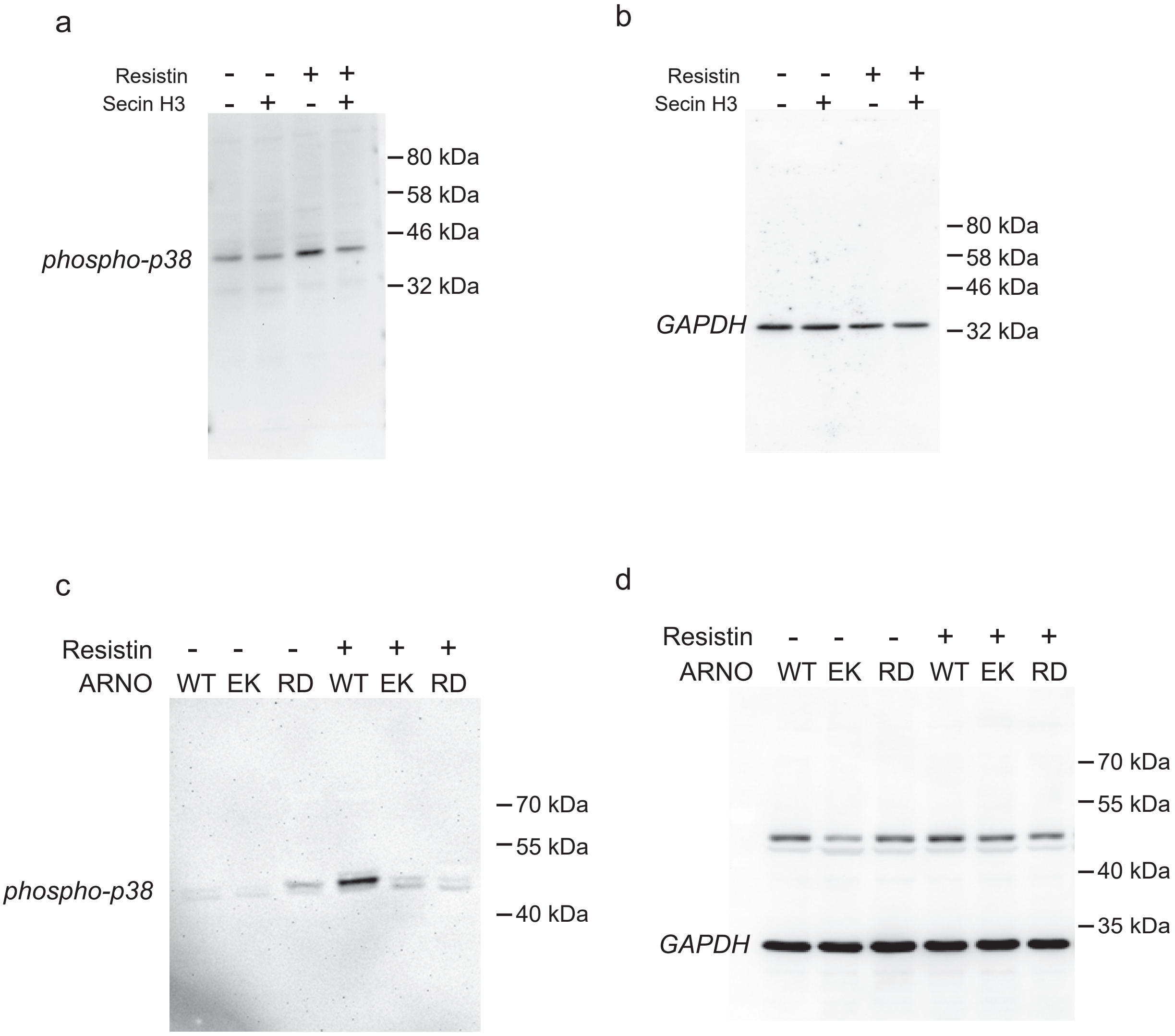

Supplementary figure 6

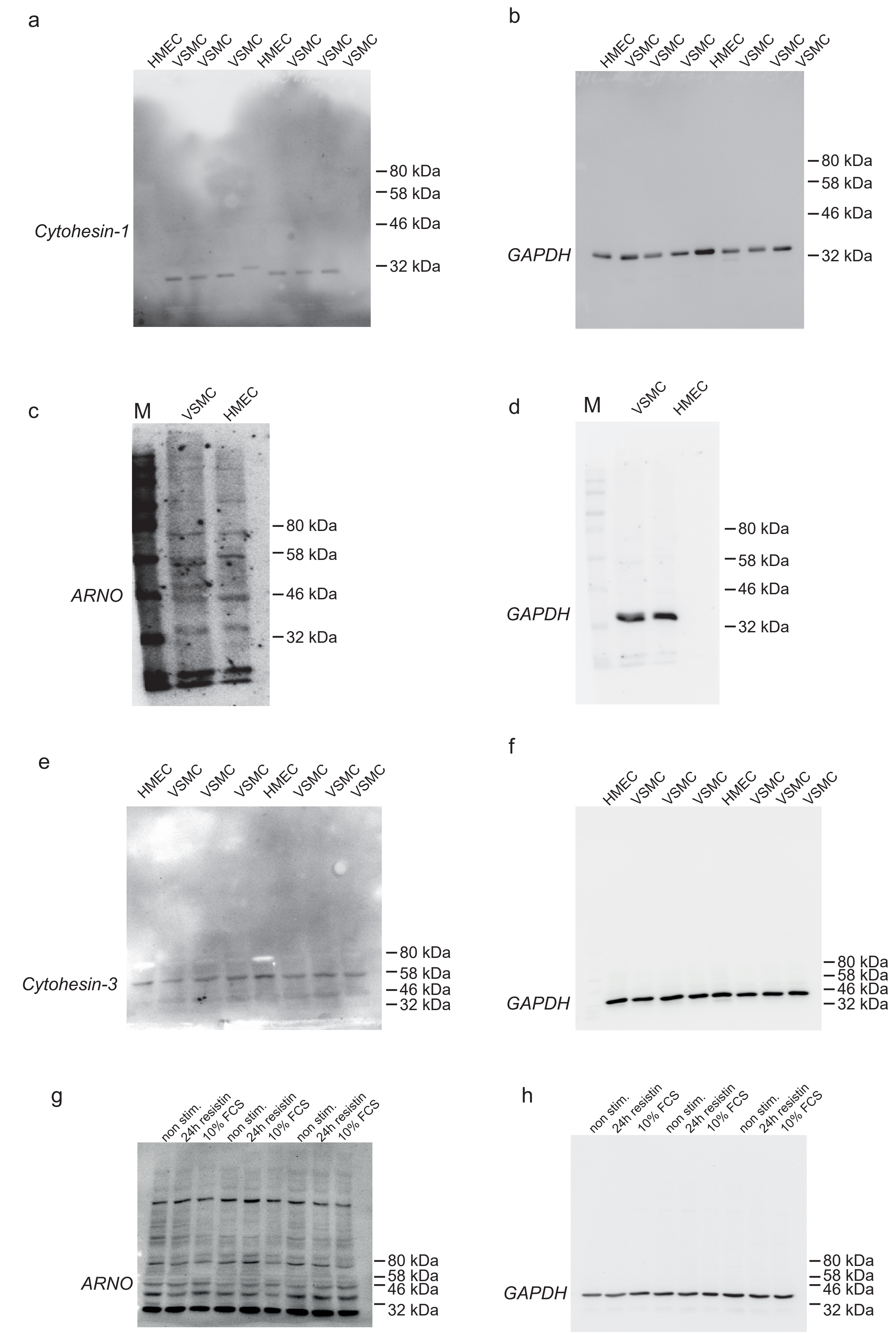

Supplement: Supplementary file 1 — Supplementary information [file 41598_2020_60446_MOESM1_ESM.pdf]
